# Combining graph neural networks and computer vision methods for cell nuclei classification in lung tissue

**DOI:** 10.1016/j.heliyon.2024.e28463

**Published:** 2024-03-22

**Authors:** Jose Pérez-Cano, Irene Sansano Valero, David Anglada-Rotger, Oscar Pina, Philippe Salembier, Ferran Marques

**Affiliations:** a Department of Signal Theory and Communications, Universitat Politècnica de Catalunya, Barcelona, Spain; b Department of Pathology, Vall d'Hebron University Hospital, Barcelona, Spain

**Keywords:** Histology, Lung cancer, Graph neural networks, Convolutional neural networks

## Abstract

The detection of tumoural cells from whole slide images is an essential task in medical diagnosis and research. In this article, we propose and analyse a novel approach that combines computer vision-based models with graph neural networks to improve the accuracy of automated tumoural cell detection in lung tissue. Our proposal leverages the inherent structure and relationships between cells in the tissue. Experimental results on our own curated dataset show that modelling the problem with graphs gives the model a clear advantage over just working at pixel level. This change in perspective provides extra information that makes it possible to improve the performance. The reduction of dimensionality that comes from working with the graph also allows us to increase the field of view with low computational requirements. Code is available at https://github.com/Jerry-Master/lung-tumour-study, models are uploaded to https://huggingface.co/Jerry-Master/Hovernet-plus-Graphs, and the dataset is published on Zenodo https://zenodo.org/doi/10.5281/zenodo.8368122.

## Highlights

• Graph-based tumoural cell detection improves over previous state of the art.
• Batch normalisation usefulness depends on graph architecture.
• Preparing a dataset of 85 images from 9 patients with lung tumour disease.
• Open-source code and pre-trained models available for reproducible research.

## 1 Introduction

Lung cancer is a prevalent type of cancer, along with breast, colon, rectum and prostate cancers. It is also the leading cause of cancer-related deaths worldwide with almost two million deaths per year [1]. The early detection of any type of cancer is vital for increasing the survival probability and the effectiveness of the applied treatments. Tissue samples obtained via biopsy are used to diagnose cancer. These samples are fixed in fomalin, embedded in paraffin, sliced and stained with Hematoxylin and Eosin (H&E) and/or special stains to easily identify the different cells and patterns of the tumour. Pathologists then analyse the slides obtained from the samples and make their final diagnosis.

Digital Pathology (DP) emerges from the digitisation of pathology slides into high-resolution images. It has revolutionised how histology related diagnosis is addressed, shifting the paradigm from the traditional microscope to software computing. Computer aided diagnosis (CAD) methods aim at employing machine learning algorithms to reduce the time expert pathologists spend manually inspecting histology slides to make a diagnosis and hence, to reduce their daily workload on repetitive tasks.

In the context of Digital Pathology, the Catalan Health Institute (Institut Català de la Salut, ICS) launched in 2020 the so-called DigiPatICS project [2]. This project aims at deploying digital pathology in an integrative way within a network of eight hospitals in Catalonia, Spain, giving service to over 168 pathologists and processing over 1 million slides each year. The project creates a network among ICS centres allowing the eight hospitals to work as one. The DigiPatICS project includes the development and sharing of image processing and deep learning tools to recognise tissue patterns, to select tumour areas, and to quantify them, among other tasks. The tools are required to cover a large range of pathologies and the project started tackling the breast and lung cancer cases. This paper presents research works done for the latter case.

In this paper, our primary application is cell nuclei classification into normal and tumoural on lung tissue. Most of the deep learning architectures that tackle this kind of problems rely on Convolution Neural Networks (CNNs). The main objective of this paper is to demonstrate that combining a CNN with a cell-based Graph Neural Network allows us to improve the final classification results. The CNN segments the cell and generates their initial classification and the Graph Neural Network post-processes this information to create an improved classification. The first tool we used is HoVer-Net [3]. It is a Convolutional Neural Network-based architecture developed for both cell segmentation and classification that has achieved state-of-the-art performance in popular benchmarks [3], [4], [5], [6], [7]. The architecture is fed with small image patches of size 270×270 pixels and simultaneously outputs the segmentation and classification maps. In practice, the field of view involved in these input patches is so small that only a low number of cells can be observed. As a result, the architecture prevents the method to capture inter-nuclei interactions information and therefore the classification essentially depends on the nuclei characteristics and narrow pixel context. However, although cell classification depends indeed on individual cell specific features, it is also influenced by the other nearby cells. Hence, we propose to employ cell graphs and a Graph Neural Network-based classifier on top of HoVer-Net to boost the classification of tumour cells on lung tissue tiles extracted from whole-slide images (WSIs) from patients with cancer diagnosis.

In this work, we have created a dataset of 85 tiles of size 1024×1024 pixels with cell level annotations extracted from 9 lung WSIs. The annotations define the cells' nuclei shape and classify each cell as either cancerous or non-cancerous. Then, we have developed an architecture sequentially composed of a Convolutional Neural Network (CNN), specifically the Hover-Net, and a Graph Neural Network (GNN) to address the cell nuclei segmentation and classification tasks. We show that this approach significantly outperforms the baseline classification obtained by using only the HoVer-Net.

The main contributions of our work can be summarised as follows:

• An incremental procedure to facilitate the annotation of WSIs at the finest granularity level.
• An annotated dataset of H&E stained lung tissue following that procedure and manually reviewed by the expert Irene Sansano Valero.
• A novel method for improving classification of tumoural cells by leveraging computer vision models and graph neural networks.
• The weights of all the models from our method trained on our dataset.
• A Python library that implements our method and many preprocessing utilities to facilitate the work of future researchers.

## 2 Related work

Deep learning techniques have been used in a wide variety of applications within the field of Digital Pathology (DP) such as cell segmentation and classification, tumour detection and subtyping, tissue phenotyping and diagnosis from gigapixel WSIs [3], [8], [9]. Due to the revolution that Convolutional Neural Networks (CNNs) caused in the vision domain, they are naturally the de facto standard to tackle DP challenges from a deep learning perspective. Nonetheless, CNNs operate on raw images, which are signals defined on the pixel space and therefore ignore the concept of biological entity such as cell, gland or tissue, which is the main subject of study of medical practitioners. Additionally, CNNs fail at capturing the hierarchical structure of the tissue and their fixed input patch size limits their ability to represent larger scale relationships.

Alternatively, graph representations of histology slides have been proposed to overcome the aforementioned CNN failure cases [10], [11], [12], [13], [14], [15], [16], [17], [18], [19]. Specifically, Cell Graphs (CGs) can be constructed by detecting with a CNN the cell nuclei in images. These cell nuclei form the nodes of the graph and can be associated with categorical information [16], [17]. The edges of the graph can be created based on nearest neighbours [12], [14] or using the Delaunay triangulation to obtain a planar graph [16], [17]. Moreover, because of their recent success for graph-structured data analysis, Graph Neural Networks (GNNs) have been applied to process these CGs. Some works choose the architecture of GraphSAGE [12], [16] while others employ the Graph Isomorphism Network (GIN) [14]. We test both on our dataset and expand the comparison to graph convolution and attention [20], [21]. Intuitively, the combination of CGs and GNNs enables both working on the biological entity space of cells and the contextualization of these entities with their environment. Indeed, GNNs fed with cell nuclei data have shown promising results in cell applications, tumour subtyping and grading from image tiles [12], [14], [17]. However, due to the difficulty of obtaining cell-level labels many works resort to WSI classification as in [12], [14] or simplify the labelling process by identifying high-level structures such as the basement membrane of the oral mucosa [16]. We extend on that corpus of research by showing that the usage of a GNN on top of a CNN can improve the original CNN classification for lung tissue. To the best of our knowledge our work is the first to apply CNN and GNN for cell classification in lung tissue on a database that was manually reviewed at each cell individually.

## 3 Material

To develop and validate our proposed architecture, we needed a dataset of lung cancer samples that were previously stained with Hematoxylin and Eosin (H&E). Our first task was to search for possible publicly available datasets of lung cancer samples with proper annotation and similar scanning process as the one that is currently implemented in the hospitals of the DigiPatICS project. Colour and intensity variations resulting from different scanning procedures among hospitals/scanning hardware represent a very significant challenge, as the performance of models trained on one database may not generalise well to other hospitals' data. Moreover, our goal was to work with a dataset that could represent a wide range of medical conditions and tissue types and that had a precise annotation at the cell level. That is, we were requiring the annotation to precisely represent each cell shape and to associate to each cell its class (cancerous or non-cancerous). As we did not find any dataset fulfilling all these requirements and were willing to develop models that had optimal performances for the staining and scanning protocols of the DigiPatICS hospitals, we decided to create a specific dataset. Note that all the DigiPatICS hospitals follow a similar staining and scanning protocol and use the same scanning devices with a predefined configuration.

The WSIs were obtained from the Vall d'Hebron Hospital in Barcelona and scanned with PANNORAMIC 1000 Flash DX scanners of 3DHISTECH with resolution 0.25μm /pixel, single layer, and 40x magnification. Working directly with WSIs is an unfeasible task due to their large size between $8\cdot 10^{4}$ and $2\cdot 10^{5}$ pixels by side, so we opted instead to work with subimages called tiles. The dataset is composed of 85 tiles of size 1024x1024 pixels. The tiles were manually extracted from WSIs of 9 different lung cancer patients. They were extracted from specific regions of interest of WSIs indicated by pathologists. The goal was to capture the widest possible variety of situations with a limited number of tiles.

This dataset had to be annotated by expert pathologists. The pathologists essentially evaluated the nucleus-cytoplasm ratio, the cell colour, the nuclei regularity, the presence of papillae acini or solid nests as well as the characteristics of nearby cells. This is a very tedious and time consuming task as tiles may involve between 100 and 1000 cells. It was felt that asking pathologists to annotate the set of tiles without any prior help was unrealistic. As a result, we used an incremental strategy relying on a combination of tools from mathematical morphology and deep learning models to efficiently produce accurate annotations for our Lung Cancer dataset.

The first step involved using a tool from mathematical morphology called the maxtree representation [22]. The use of the maxtree allowed us to efficiently detect the shape of candidate cells, to study the colour distribution of the pixels belonging to these cells as well as to extract geometrical features (size, elongation, etc.). By manually setting threshold values on the colour and the geometrical features, we were able to generate an initial annotation. To improve the quality of the annotations, we relied on the help of an expert pathologist from the Vall d'Hebron Hospital: Irene Sansano Valero. The pathologist reviewed and corrected the whole initial annotation. This work was still very time consuming but the fact that preliminary cell candidates were already defined and classified was an important help. The pathologist had to either reject erroneous cells or create the missing ones. Moreover, if necessary, she had to correct the class assignment (cancerous and non-cancerous). The annotations correction itself was done using the QuPath [23] software which is a cross-platform tool for digital pathology image analysis.

With this initial corrected annotation, we trained a deep learning model, specifically a HoVer-Net model, to predict annotations over the entire set of images in the dataset. This process allowed us to produce again a set of images with a preliminary annotation but this time over the entire dataset. This preliminary annotation was again corrected and improved with the help of the pathologist to ensure its accuracy.

By following this process, we have been able to produce the annotations for the mentioned 85 tiles. The use of morphological tools in an initial phase, followed by the predictions of a deep learning model in a more advanced annotation phase, proved to be effective in reducing the workload of the pathologist and in improving the accuracy of the annotations. At the end, the dataset includes 85 tiles involving 8360 cancerous cells, 22542 non-cancerous cells and 153 uncertain cells. The tiles themselves represent a wide variability of lung tissues and cells. They contain cartilages, macrophages, erythrocytes, lymphocytes, inflammatory and necrotic cells among others. All cells were initially classified as either healthy or tumoural based on the criterion of the expert although, in some cases there was not enough information to decide and those cells were classified as unknown. Finally, the train / validation/ test partition was made based on whether the patient did not have any single unknown cell. Those patients where the doctor was sure about the label of every cell were included in the test set.

## 4 Method

**Figure 1:**
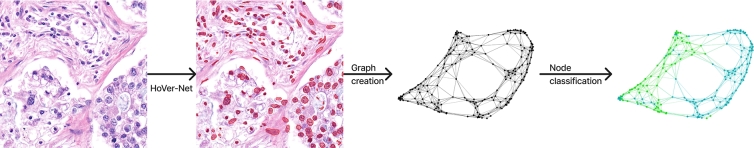
Overview of our method. An initial segmentation and classification of the cells is obtained using HoVer-Net. Then, we create a graph using the cells as nodes and apply graph neural networks to improve the classification made by HoVer-Net using the relationships between nodes.

An overview of our proposal can be seen in Fig. 1. This figure shows how the original image is initially processed by the computer vision method, HoVer-Net. This CNN segments the cells and defines their shapes and classes. Then, a graph is formed by associating each cell to a graph node. The graph topology is defined through heuristic rules. The GNN itself is used in the last step to take into account the interaction between cells and to improve their classification. In this section, we discuss the details of the proposed processing approach, together with some necessary background to understand it. Specifically, subsection 4.1 defines the problem and expresses the main notation. Then, subsection 4.2 and subsection 4.3 describe the two stages of our proposed method, the first subsection focuses on the computer vision approach and the second one on the graph creation and processing.

### 4.1 Problem formulation

We denote the training data by $D=\{(X_{i},t_{i}):i\in [N]\}$ where *N* is the number of different samples, $X_{i}$ are histological images and $t_{i}$ are the target values we are interested in predicting from $X_{i}$. These target values can be referred to all elements in the image, and therefore be pixel classes, or to the nuclei detected in the image, and therefore be cell classes. In our problem, the classification of the nuclei is more important than their segmentation since physicians require a percentage of tumoural nuclei, without considering any cell-level information like the area or the perimeter of the cell. For that reason, we will refer to $t_{i}$ equally as images and as sets of nuclei $t_{i}=\{(x_{j}^{i},y_{j}^{i},c_{j}^{i}):j\in [N_{i}]\}$, each of them represented by their coordinates $\left(x_{j}^{i},y_{j}^{i}\right)$ and class $c_{j}^{i}$. Each image can contain a different number of cells $N_{i}$. When dealing with cell classification we consider a binary classification problem $c_{j}^{i}\in \{0,1\}$, where the label 0 means a healthy cell and the label 1 means a tumoural cell. When considering $t_{i}$ as an image, thus being a pixel-wise classification problem, each pixel can have value 0, 1 or 2 meaning background, non tumour and tumour respectively.

### 4.2 The image processing module

Convolutional Neural Networks (CNNs) are widely adopted in the field of image processing. However, training a simple CNN to predict the pixel labels $t_{i}$ from $X_{i}$ commonly does not manage to fully split nearby instances and often merges neighbouring cells into large connected components [24], [25], [26]. As a result, postprocessing algorithms are often applied to the CNN raw predictions. A typical solution involves the watershed algorithm [27]. We selected a state of the art CNN approach, the HoVer-Net [3], as the first stage of our method.

#### 4.2.1 The HoVer-Net three branches

To tackle the challenge of improving the cell segmentation and classification, the HoVer-Net involves three branches. To maintain the nomenclature of the HoVer-Net article [3] we will refer to the branch that predicts the raw class of each pixel as the type prediction (TP) branch. The second branch distinguishes the cells from the background and it is referred to as the nuclear prediction (NP) branch. The last branch predicts the horizontal and vertical distances from each pixel to the centroid of the cell and is named the horizontal and vertical branch (HV).

**Figure 2:**
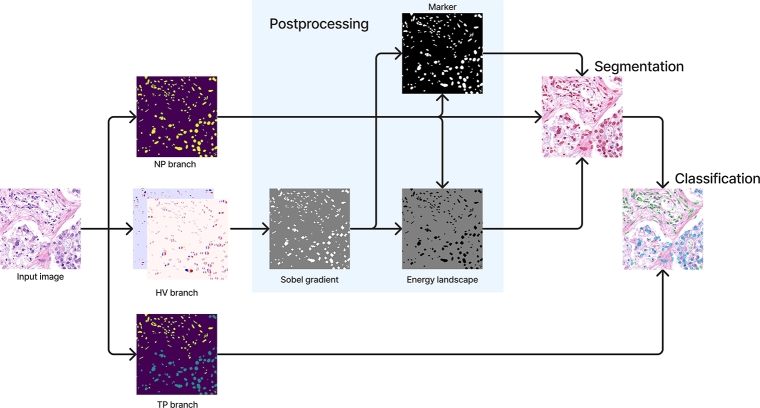
High level overview of the HoVer-Net computer vision approach. Three different predictions are made. The NP and HV branches are combined using the watershed algorithm to create the segmentation and finally the labels are obtained using majority voting between the segmentation and the TP branch.

The process is visualized in Fig. 2. Internally each branch is composed by an encoder-decoder architecture. The encoder is composed of residual networks with downsampling while the decoder is composed of convolutional and dense networks with upsampling. One key aspect of this approach is that the encoder is shared by all three branches, while each branch has a different decoder. This enables the network to learn useful features for all three tasks at the same time.

#### 4.2.2 Loss function

The loss function used to train the HoVer-Net is a linear combination of two different losses per branch, six losses in total. For the TP branch and the NP branch the binary cross-entropy and the dice loss are used [28], while the HV branch uses the mean square error [29] and the mean square gradient error [3]. Formally,

(1) $$L=\lambda_{bce}^{TP}L_{bce}^{TP}+\lambda_{dice}^{TP}L_{dice}^{TP}+\lambda_{bce}^{NP}L_{bce}^{NP}+\lambda_{dice}^{NP}L_{dice}^{NP}+\lambda_{mse}^{HV}L_{mse}^{HV}+\lambda_{msge}^{HV}L_{msge}^{HV}$$

Where $\lambda_{bce}^{TP},\lambda_{dice}^{TP},\lambda_{bce}^{NP},\lambda_{dice}^{NP},\lambda_{mse}^{HV},\lambda_{msge}^{HV}$ are hyperparameters. Using this loss function, the three branches are trained simultaneously.

#### 4.2.3 Postprocessing

When the three branches are trained, the output of the NP and HV branches are combined to create the cell segmentations. To decide the label of each cell, the segmentation result is combined with the TP branch output; that is, the class of a cell is predicted by taking the majority voting of the pixel predicted types inside the cell.

More concretely, the watershed algorithm [30] is used to improve the result of the NP branch. The algorithm requires an energy landscape and a marker. The energy is defined as follows

(2) $$E=(1-S_{m}(X))⊙f^{NP}(X)$$

where ⊙ denotes element-wise multiplication and $S_{m}(X)=\max(H_{x}⁎f_{x}^{HV}(X),H_{y}⁎f_{y}^{HV}(X))$ is an estimation of the gradient magnitude of the HV branch applying horizontal and vertical Sobel operators to the horizontal and vertical components of the HV branch respectively. In turn, the marker is defined as

(3) $$M=\text{ReLU}(f^{NP}(X)-S_{m}(X))$$

Based on empirical results, $f^{NP}$ and $S_{m}$ were thresholded prior to computing the energy and the marker images [3]. Finally, *M* and *E* are used as the marker and energy landscape respectively to split $f^{NP}(X)$ with the watershed algorithm.

As previously commented, once the segmentations have been computed, the output of TP determines the class of each cell by taking the majority vote. For each cell, we also estimate the probability of the given class by taking the proportion of pixels with the majority label with respect to the other pixels in the cell that are not classified as background by the TP branch. This is to ensure that the probability of the predicted class is always greater than 0.5 since we are dealing with a binary classification problem. Otherwise, after thresholding the prediction, incoherent results may appear.

### 4.3 The graph processing module

Now that the cells segmentation task has been described, this subsection explains how to formulate the problem as a node classification problem. subsubsection 4.3.1 discusses how to define a graph from a patch. Later, the graph neural network architectures used here for node classification are described in subsubsection 4.3.2 and subsubsection 4.3.3. We have chosen them for their popularity and good performance, but notice that there are many other possible architectures that could be used.

#### 4.3.1 From images to graphs

Once cells are defined and localized, they can be interpreted as nodes in a graph and they can be connected based on their distances. This approach has its intuition on how expert pathologists diagnose: the criteria for telling if a cell is tumoural or not is based not only on the cell information itself but on that of the cells in its surroundings.

**Figure 3:**
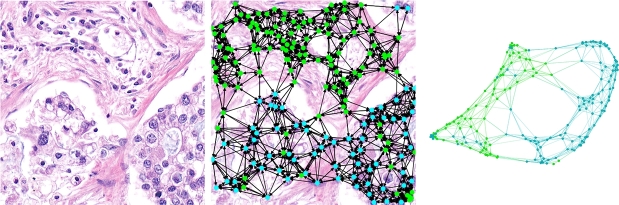
Example of an image and its associated graph. At the middle we have the nodes located at their corresponding centroids. However, a graph is an abstraction, so it may also be viewed as in the right image, since it only encodes relationships, not absolute positions. Visualization of the graph was made using Gephi [31].

Formally, we denote a graph by $G_{i}=(V_{i},E_{i})$ where $V_{i}$ is the set of nodes associated with the image $X_{i}$ and $E_{i}$ the set of associated edges. Nodes are further identified by their coordinates and a series of attributes $h_{j}$: $V_{i}=\{(x_{j},y_{j},h_{j})\in R^{2}\times R^{d}:j\in [N_{i}]\}$. In turn, the edges are constructed based on a distance threshold and limiting the degree of the nodes: $E_{i}=\{(j_{1},j_{2})\in [N_{i}]\times [N_{i}]:{\left\|(x_{j_{1}},y_{j_{1}})-(x_{j_{2}},y_{j_{2}})\right\|}_{2}\leq D,j_{2}\in N_{j_{1}}^{M}\}$. Where $N_{j_{1}}^{M}$ are the *M* closest cells to node $j_{1}$. The number of edges resulting from this procedure is linear in the number of nodes: $\left|E_{i}|\in O(|V_{i}|\right)$. In the experiments reported in section 5, the distance threshold was set to 200 pixels and the maximum node degree was 10. These values were used so that the graphs only involve one connected component and are almost planar (that is, with Euler characteristic close to 2.) Fig. 3 shows an example of such a graph construction. Note that the graph is undirected.

It remains to clarify how the attributes $h_{j}$ are extracted. Given a cell, several handcrafted features are computed from the cell contour and its interior. The features we have used are (i) the area of the cell, measured in pixels, (ii) the perimeter of the contour, also measured in pixels, (iii) the standard deviation of the pixel gray level values, and (iv) the RGB histogram quantized into 5 bins for each channel. In order to use the predictions of the HoVer-Net TP branch, the probability as described in subsubsection 4.2.3 is also included. The decision of using such features is mainly driven by their simplicity and computational efficiency. We thought about using some internal activations of HoVer-Net as extracted features, but HoVer-Net is not trained specifically to extract features of each of the cells. It works at pixel level, therefore, to extract features from HoVer-Net activations we would need to train in an end to end manner so that the extracted features are learned for each cell. A possible solution would be to use a specific CNN for this task or even to add a new branch in HoVer-Net. Moreover, classical computer vision features such as SIFT, SURF or BRIEF could also be used. These areas of improvement are left as future work.

#### 4.3.2 Graph convolutional networks

Graph convolutional networks are a type of propagation module [32] which computes the forward pass as an average of the nearby nodes. As expressed in [20] the forward pass of a single layer of the graph convolutional network can be defined as

(4) $$h_{j}^{\left(l+1\right)}=\sigma \left(b^{\left(l\right)}+\sum_{k\in N_{j}}\frac{1}{c_{jk}}W^{\left(l\right)}h_{k}^{\left(l\right)}\right)$$

where $b^{\left(l\right)}\in R^{d},W^{\left(l\right)}\in R^{d\times d}$ are the bias and weights of the layer, $N_{j}$ is the set of neighbours of node *j*, $c_{jk}=\sqrt{\left|N_{j}|\cdot |N_{k}\right|}$ is a normalisation factor and *σ* is an activation function. The vectors $h_{k}^{\left(l\right)}$ are the hidden embeddings of the network for each layer, being $h_{k}^{\left(0\right)}=h_{k}$ the initial vector of attributes defined in subsubsection 4.3.1. In the last layer, the weight matrix is of dimensions 2×d, the bias is a scalar instead of a vector and the activation function is the softmax, since we are dealing with a classification problem.

That describes one layer and to construct the full model we stack several layers. In addition, we use dropout and batch normalisation after each layer activation. Since that introduces several hyperparameters, we perform a grid search and choose the set of values with the best result in the validation set. The reader can find more details in subsection 5.3. In all cases, the cross-entropy loss was used for the optimization.

#### 4.3.3 Graph attention networks

Similar to the graph convolutional network, the graph attention network is also a propagation module [32]. The difference is that the weights are computed using the attention mechanism [33]. Mathematically, it was described in [21] as

(5) $$h_{j}^{\left(l+1\right)}=\sigma \left(\sum_{k\in N_{j}}\alpha_{jk}W^{\left(l\right)}h_{k}^{\left(l\right)}\right)$$

where $W^{l}\in R^{d\times d}$ are the layer weights and $\alpha_{jk}\in R$ are the attention weights which are defined by the following formula

(6) $$\alpha_{jk}=\frac{\exp(\text{LeakyReLU}(a\cdot [Wh_{j}||Wh_{k}]))}{\sum_{r\in N_{j}}\exp(\text{LeakyReLU}(a\cdot [Wh_{j}||Wh_{r}]))}$$

being $a\in R^{2d^{\prime }},W\in R^{d^{\prime }\times d}$ two learnable projection matrices and || the concatenation operation. The similarity function $a\cdot [Wh_{j}||Wh_{k}]$ can be computed in a space with a different dimensionality $d^{\prime }$ from the embedding space dimensionality *d*. Inspired by the multi-head attention mechanism proposed in [34], the previous attention mechanism can be extended to *H* heads(7)
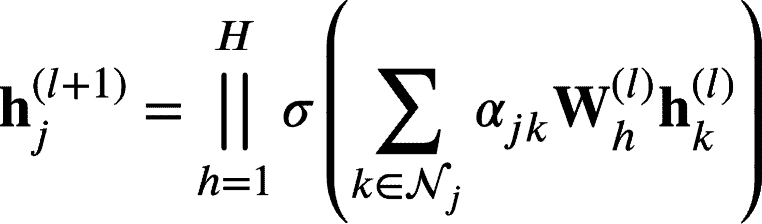
 where now $W_{h}^{\left(l\right)}\in R^{d\times Hd}$ and in the final layer heads are averaged instead of concatenated, as explained in [21]. Again, as with the graph convolution we use several layers, dropout and batch normalisation. We do not optimise the number of heads as it would exponentially increase the number of configurations to train.

## 5 Experiments

### 5.1 Evaluation metrics

Prior to benchmarking our method we need to address one problem. Segmentation tasks are normally evaluated using pixel-wise metrics like the Dice score. However, physicians are interested in counting cells, not pixels. For that reason, our metrics should reflect that. The problem comes when the prediction contains cells that were not in the ground truth (GT) or when it misses some important cells. Depending on whether we take into account those cells we get two different sets of metrics. One that evaluates the quality of the classified cells by ignoring missing cells (without missing cells) and another that tells us how good the classification is by acknowledging them (with missing cells).

For the classification metrics without missing cells to be properly computed we have to establish a mechanism for linking every GT cell with a unique predicted cell. Since the prediction may not be perfect, all the pixels may not be exactly the same as in the GT. For that reason we look at the distance between the centroids of the cells. We match a pair of GT $n_{j}=(x_{j},y_{j},h_{j})$ and prediction ${\overset{ˆ}{n}}_{j}=({\overset{ˆ}{x}}_{j},{\overset{ˆ}{y}}_{j},{\overset{ˆ}{h}}_{j})$ cells only if $n_{j}$ is the closest GT cell to ${\overset{ˆ}{n}}_{j}$ and vice versa. We will refer to these pairings as 1-1 matchings. These 1-1 matchings are efficiently computed using KD-trees [35].

For the classification metrics with missing cells we construct the confusion matrix from the 1-1 matchings and add one row and one column corresponding to an extra class, the background class. The column shows how many cells were missing and the row how many extra cells are predicted that should not be there. Note that the use of a GNN cannot change that extra column but it can modify the extra row, as it will be further discussed in subsection 5.4.

Finally, given the 1-1 matchings and the extended confusion matrix, both types of classification metrics are computed to fairly compare the different networks. We consider the F1 score (F1), accuracy (ACC) and the area under the ROC curve (AUC) because of their extended usage as binary classification metrics, since we only have healthy and tumoural cells. Apart from that we include an additional metric: the error percentage (% ERR). The error percentage is the absolute difference between the predicted percentage of tumoural cells and the real percentage. This metric is computed for both classification metrics and, in the case with missing cells, it ignores cells that do not belong to a 1-1 matching. We include this specific metric because it is the main metric of interest for our expert pathologists. For the classification metrics without missing cells we compute the Macro, Weighted and Micro F1 score using the confusion matrix, since we are trying to assess that our method improves in several aspects.

### 5.2 Performance evaluation

The result of the comparison can be seen in Table 1. We split the dataset into training, validation and test subsets (60% / 20% / 20%). The validation set is used for tuning the hyperparameters and the test set for evaluating the best validation model. The metrics are reported from the test dataset only and the analysis in the next section is done on the validation set. The test set was chosen so that it included patients in which the expert pathologist was most certain of the labels. And all the patches for each patient only appeared in one split. Our method clearly outperforms the HoVer-Net baseline showing it has benefited from formulating the problem as a node classification problem. The attention mechanism does not perform so well and, by looking at the validation metrics that are available in the sequel, it is clear that the model is overfitting.

**Table 1:** Comparison of our method with the HoVer-Net baseline on the test set. Upper table: Classification metrics without missing cells. Lower table: Classification metrics with missing cells. The best result is highlighted in bold. The results shown here come from the configuration that performed best in the validation set. Convolutional architecture is called GCN, attention mechanism is ATT, graph isomorphism network is GIN and graphsage is SAGE. The metrics in the ATT model correspond to a constant prediction.

	F1 (↑)	Acc (↑)	AUC (↑)	% ERR (↓)
HoVer-Net	57.69%	82.39%	70.48%	11.89%
HoVer-Net+GCN	**71.70%**	**85.15%**	**80.44%**	**1.04%**
HoVer-Net+ATT	0%	73.24%	50%	26.76%
HoVer-Net+GIN	70.14%	84.85%	79.02%	2.78%
HoVer-Net+SAGE	61.40%	81.73%	73.02%	6.18%



	Macro F1 (↑)	Weighted F1 (↑)	Micro F1 (↑)	% ERR (↓)
HoVer-Net	66.06%	72.11%	66.12%	10.22%
HoVer-Net+GCN	**72.54%**	**75.88%**	**68.33%**	**0.87%**
HoVer-Net+ATT	44.79%	37.19%	58.76%	25.00%
HoVer-Net+GIN	71.86%	75.48%	68.09%	0.96%
HoVer-Net+SAGE	67.18%	72.31%	65.59%	4.49%

In order to try and compare with previous methods we also tested GraphSAGE [36] and GIN [37] since they are the architectures used in other similar articles that apply GNNs on medical problems. We could not compare directly with those methods because they were used for different problems, like WSI classification instead of cell classification, and because their datasets are not public. For those reasons we provided our own implementation and evaluated on our own dataset. Our results suggest that the convolutional architecture outperforms the GraphSAGE architecture while being similar to the graph isomorphism network. In all the cases except for the attention network the GNNs are proven to be beneficial over simply using HoVer-Net.

### 5.3 Hyperparameter analysis

**Figure 4:**
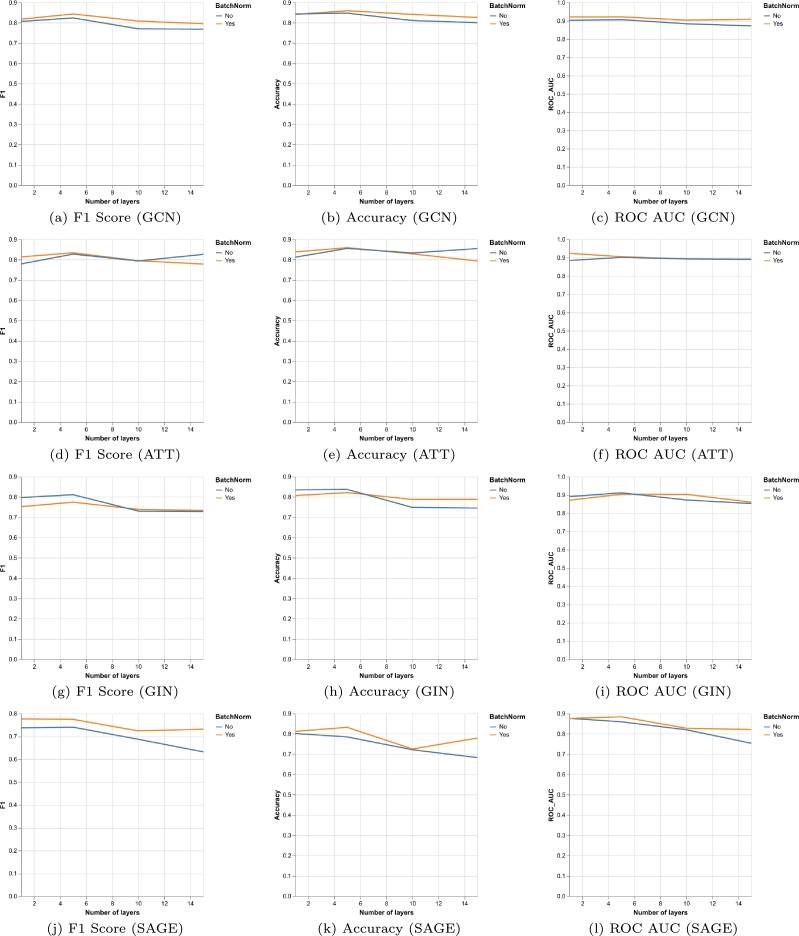
Comparison of the effect that applying batch normalisation has on the result for the four graph architectures on the validation set. Plots show the maximum value that was obtained on the validation set across all the different dropout ratios. X-axis shows the number of layers of the model.

**Figure 5:**
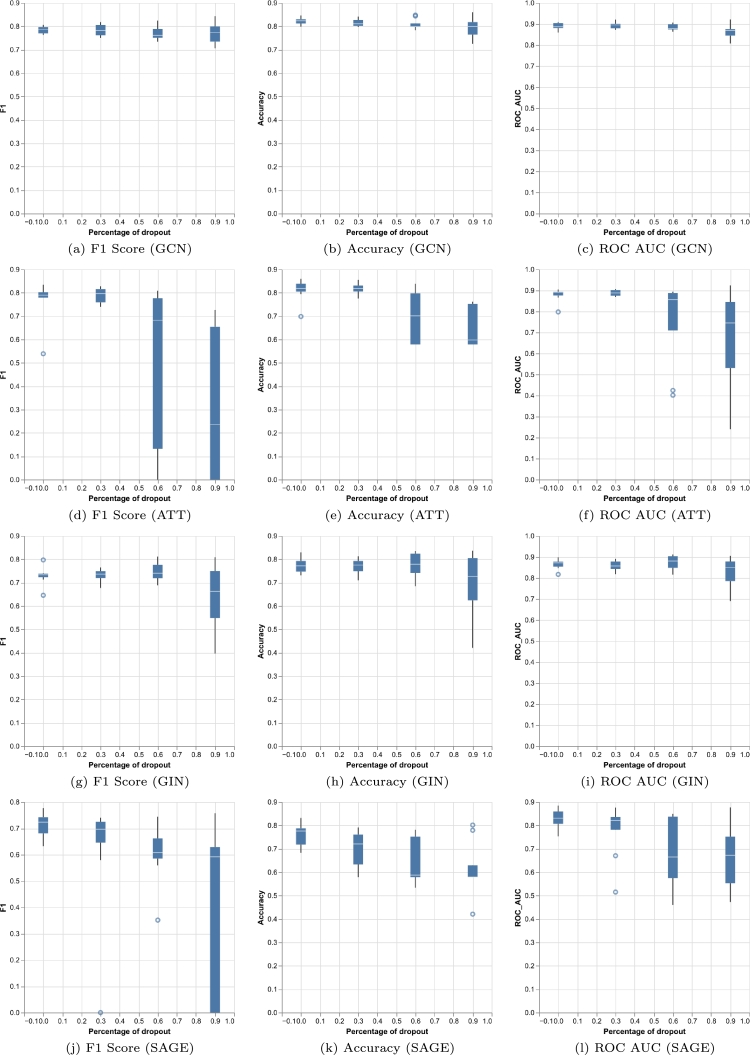
Comparison of the effect that different dropout ratios have on the result for the four graph architectures on the validation set. X-axis shows the different dropout ratios. The box plots display the variance of the model with respect to the other hyperparameters, which are the number of layers and the usage of batch normalisation.

The exact graph neural network architecture depends on three hyperparameters: number of layers, dropout ratio and batch normalisation. We performed a grid search on the validation set to obtain the best configuration. The result of such optimisation is visualised in Fig. 4 and Fig. 5. We can observe in Fig. 4 that the usage of batch normalisation after each layer activation is only beneficial for the convolutional and GraphSAGE architectures and not always for the attention and GIN mechanisms. However, the impact of batch normalisation is not decisive on the result, it only slightly alters the performance. In Fig. 5 we can also see the variance of the model when using different dropout ratios. In the convolutional architecture dropout does not have a big effect, but in the attention architecture an excessive amount of dropout can cause a large drop in performance similar to what happens with GraphSAGE. The GIN architecture lies in the middle, it is affected but not drastically. Graph convolution performs well for all the different combinations of hyperparameters but the other networks can be more difficult to train appropriately. Overall the best configurations were

• Number of layers: 5 (GCN), 15 (ATT), 5 (GIN), 1 (SAGE)
• Dropout ratio: 0.9 (GCN), 0 (ATT), 0.6 (GIN), 0 (SAGE)
• Batch normalisation: True (GCN), False (ATT), False (GIN), True (SAGE)

### 5.4 Correcting segmentation

We have shown that by stacking a GNN on top of HoVer-Net we can improve the classification of the cells. In theory, it is also possible to improve the segmentation. Although it is impossible to generate new cells we can train an extra head in the last layer to predict if a given cell is real or not. As mentioned in subsection 5.1 we can modify the first row of the confusion matrix corresponding to extra cells but not the first column corresponding to missing cells. This way we can eliminate cells that were predicted by HoVer-Net but in reality do not exist. The result of such experiment is on Table 2. Unfortunately our approach did not work. The problem here is that the extra head added is trying to solve a highly imbalanced problem. There are very few extra cells that need to be removed. Since we are training the two heads simultaneously, the head in charge of the classification ends up dominating the training and the head in charge of detecting extra cells just predicts every cell as a valid one. For that reason the result is just slightly worse.

**Table 2:** Result of training an extra head to correct cells predicted by HoVer-Net that do not exist. The GCN trained with that extra head is called 2-heads. Left table: Classification metrics without missing cells. Right table: Classification metrics with missing cells.

	F1 (↑)	Acc (↑)	AUC (↑)	% ERR (↓)
HoVer-Net	57.69%	82.39%	70.48%	11.89%
HoVer-Net+GCN	**71.70%**	**85.15%**	**80.44%**	**1.04%**
2-heads	66.95%	84.29%	76.41%	5.98%



	Macro F1 (↑)	Weighted F1 (↑)	Micro F1 (↑)	ERR (↓)
HoVer-Net	66.06%	72.11%	66.12%	10.22%
HoVer-Net+GCN	**72.54%**	**75.88%**	**68.33%**	**0.87%**
2-heads	70.34%	74.70%	67.64%	3.96%

### 5.5 Computational cost

All the experiments were carried out using a NVIDIA RTX 3090 GPU. The experiments on Table 1 took 3 hours and 48 minutes to complete. The majority of the computation comes from training the HoVer-Net backbone and from trying many different graph configurations. Note however that training a single GNN configuration takes just a few minutes. The reduction of dimensionality that comes from only working with node features makes the algorithm more memory and computationally efficient. With 24GB of RAM the HoVer-Net could only be trained by dividing the 1024 by 1024 patches into even smaller images. However, we could train 32 GNNs at the same time in parallel with the whole graph without needing to split it into subgraphs. This makes our method more scalable than previous convolutional neural network based algorithms.

## 6 Discussion

### 6.1 Qualitative study

**Figure 6:**
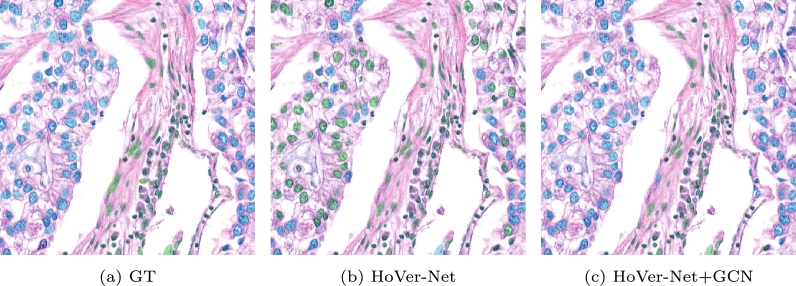
Comparison of the result of our method with respect to the HoVer-Net baseline and the ground truth. Cell classes are blended into the image. Blue means tumoural and green means healthy.

Graph neural networks introduce a different inductive bias to the problem, this means that the model has a different type of invariant and, therefore, it is taking advantage of a different symmetry. The purpose of this section is to illustrate how that bias is affecting the solution. By modelling the problem with a graph and applying a message passing network to it, we are implicitly assuming one hypothesis. We are assuming that if a cell lives surrounded by tumoural cells it is quite likely that it is also a tumoural cell. A method that takes that hypothesis into account should try to form groups of cells and not predict any isolated cell; that is, a healthy cell surrounded by tumoural cells of vice versa. In Fig. 6 we can see the effect of using GNNs. In that image there are three clearly separated groups of cells. HoVer-Net does not internally model that structure and simply tries to classify each pixel based on their surroundings. The GCN on the other hand operates directly over the graph. That graph contains three subgraphs where cells in a given subgraph are more connected among them than with other cells outside this subgraph. That piece of information gives the model the advantage to better classify the cells.

**Figure 7:**
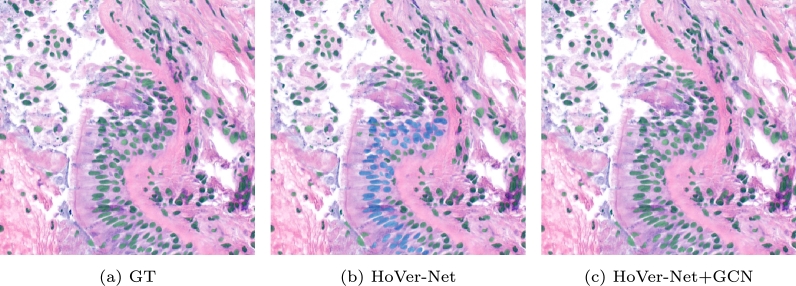
Comparison of the results in a patch containing a cilium. The cilium is the thin vertical line that is located at the left of the biggest group of cells.

**Figure 8:**
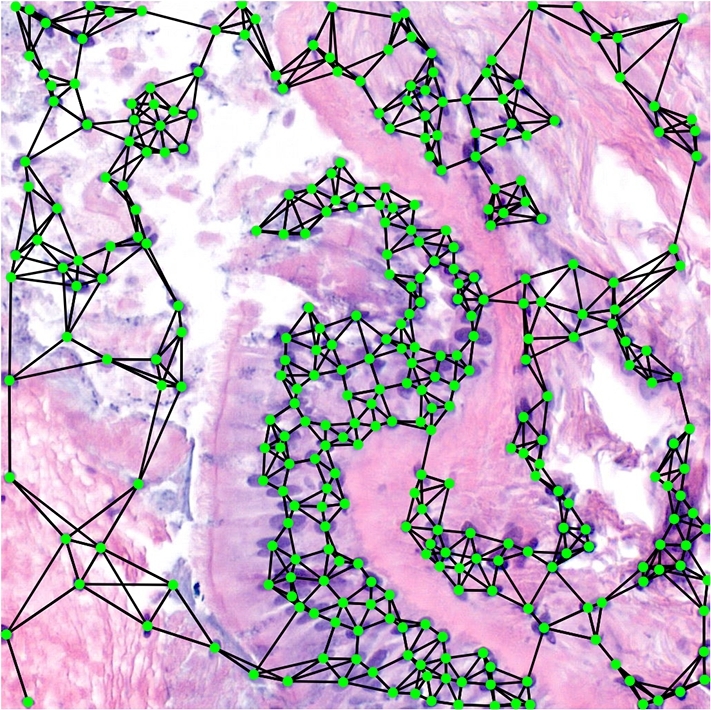
Patch containing a cilium. It has the graph representation overlayed on top. The cilium is the vertical purple filament at the centre left of the image.

A second example involving cilium can be seen in Fig. 7. The cilium is a filament that mediates the critical function of mucociliary clearance, cleansing the airways of inhaled particles and pathogens [38]. This structure is specially difficult to classify by HoVer-Net because the cells are visually very similar to other tumoural cells. The reason they are healthy is because of the presence of the cilium. HoVer-Net misclassifies them because it misses contextual information. Fig. 8 illustrates why the GCN has an advantage in this situation. We can see that the free space left by the bronchiole (the white space) has created a particular graph structure. Even though the cilium itself is not present in the graph directly, it interacts with the rest of the tissue affecting the graph topology indirectly. Therefore, the graph provides more information than the initial pixel-level analysis of the image, and therefore the GNN can better classify the cells.

## 7 Conclusions

In this paper we have introduced a new way of modelling the cell nuclei classification problem for lung tissue. We have validated the idea that the property of being a tumoural cell highly depends on the surrounding cells and shown that our method outperforms previous state of the art models. However, not every graph architecture performs equally well. Graph convolutions work well independently from the hyperparameters chosen while the attention mechanism tends to overfit.

For the sake of reproducibility we provide the source code used to obtain the results, which is available on GitHub, the pretrained models which are uploaded to Hugging Face and the dataset which is uploaded at Zenodo.

As future work it remains to find an efficient way to train everything end to end. Some of the challenges to overcome are the efficiency of running the CNN per each cell at each iteration and how to backpropagate the gradient over the intermediate morphological steps. One possible approach could be to parallelise the computation using techniques similar to that of anchor points and to approximate the morphological operations by some differentiable functions. It is also left as future work the problem of training an extra head of the graph network to remove extra cells predicted by the backbone segmentation model.

Finally, we would like to mention that it is worthwhile investigating the application of this approach of cell nuclei segmentation and classification to other types of cancer.

## Funding

This project was funded by 10.13039/501100008530European Regional Development Fund, Programa operatiu FEDER de Catalunya 2014-2020, SA18-014623 DIGIPATICS. UPC activity in this project has been partially supported by PID2020-116907RB-I00, funded by MCIN/AEI/10.13039/501100011033 and by the Spanish Research Agency (10.13039/501100011033AEI) under project PID2020-116907RB-I00 of the call MCIN/ AEI /10.13039/50110001103.

## CRediT authorship contribution statement

**Jose Pérez-Cano:** Conceptualization, Data curation, Formal analysis, Investigation, Methodology, Software, Visualization, Writing – original draft, Writing – review & editing. **Irene Sansano Valero:** Data curation. **David Anglada-Rotger:** Conceptualization, Resources, Writing – original draft. **Oscar Pina:** Formal analysis, Investigation. **Philippe Salembier:** Project administration, Writing – original draft, Writing – review & editing. **Ferran Marques:** Funding acquisition, Project administration, Writing – original draft, Writing – review & editing.

## Declaration of Competing Interest

The authors declare that they have no known competing financial interests or personal relationships that could have appeared to influence the work reported in this paper.
